# EDITORIAL: Eating disorders in diabetes: Discussion on issues relevant to type 1 diabetes and an overview of the Journal’s special issue 

**DOI:** 10.1186/s40337-019-0256-0

**Published:** 2019-07-18

**Authors:** Ann Goebel-Fabbri, Paul Copeland, Stephen Touyz, Phillipa Hay

**Affiliations:** 1 1101 Beacon St. Suite 8, West Brookline, MA 02446 USA; 2 Endocrine Unit and MGH Weight Center, Massachusetts General Hospital, and Harvard Medical School, Boston, MA USA; 3 School of Psychology, Faculty of Science, the University of Sydney, Camperdown, New South Wales Australia; 4 InsideOut Institute, Charles Perkins Centre, University of Sydney, Sydney, Australia; 5 Translational Health Research Institute, School of Medicine, Western Sydney University, Sydney, NSW Australia

**Keywords:** Disordered eating behavior, Eating disorders, Type 1 diabetes, Insulin restriction

Research suggests that women with Type 1 diabetes mellitus (T1DM) have close to 2.5 times the risk for developing an eating disorder compared to women without T1DM [1]. Women with T1DM can present with the full range of eating disordered symptoms however, the majority of research is focused only on those involving insulin restriction as a weight control behavior. It is unclear why girls and women with T1DM have increased rates of disordered eating behaviors and diagnosed eating disorders, but T1DM is strongly associated with a number of common eating disorder risk factors. For example, people with diabetes have twice the risk of clinically significant depression than those without diabetes [2]. Women and girls with T1DM also often have a higher BMI than their peers without diabetes [3]. Far less is known about Type 2 Diabetes (T2DM) and eating disorders [4] but management can be similarly challenging when it is comorbid with an eating disorder.

Other aspects of diabetes treatment may also increase the risk of eating disorders. The treatment itself involves paying close attention to refined carbohydrates and to food portions which can parallel the rigid thinking about food, weight, and body image reported by women with eating disorders who do not have diabetes [5]. Such treatment recommendations can lead to feelings of deprivation, resentment and shame, and to binge eating. Studies have found that disturbed eating behaviors in T1DM are strongly predicted by higher Body Mass Index (BMI), higher shape and weight concerns, lower self-esteem, and depressed mood. Positive feelings about appearance, the absence of depression, and a lower BMI may be protective factors [6–9]. Higher diabetes-related family conflict also appears to be a risk factor [10]. Notably, adolescence is a time of increased risk for both eating disorders and for worsening of glycemic control. The latter could reflect metabolic changes during this time, and as well it is the period when responsibility for insulin administration transitions from the parent(s) to the child.

Women with T1DM and eating disorders have A1c values approximately 2 or more percentage points higher than similarly aged women with T1DM without eating disorders. (The A1c is a laboratory test that estimates the average blood glucose values over a three-month period.) Patients who restrict insulin as a purging behaviour have higher rates of hospital and emergency room visits, higher rates of medical complications, and more negative attitudes toward T1DM than women who do not report insulin restriction [11–13]. Endorsing just insulin restriction alone was shown to increase mortality risk 3-fold over an 11-year period [14]. Even lower threshold disordered eating behaviors are strongly associated with significant medical and psychological consequences [15]. Although current treatment encourages a goal A1c of 7% or below, this target can seem unattainable and lead to disengagement from self-management of T1DM. Alternatively, diabetes treatment goals can also encourage perfectionism and lead to frustration, because blood glucose cannot be kept in range at all times.

Diabetes specialists report feeling frustrated by the dearth of specialized treatment programs for eating disorders in people with T1DM [16]. T1DM patients with eating disorders are more likely to drop out of treatment and also show worse outcomes with conventional outpatient treatment for eating disorders [17, 18]. Longer stays in residential treatment are reportedly associated with better outcomes, perhaps highlighting greater complexity and need in this population [19]. Taken together, this information underscores the need for effective treatments for eating disorders in T1DM. Current treatment guidelines are helpful but limited as they are based upon clinical expertise rather than rigorous research [20–22].

This special issue of the Journal of Eating Disorders addresses vital gaps in the research literature. At the time of this Editorial the following papers were published. Abbott and colleagues conducted a systematic review of Binge Eating Disorder (BED) and Night Eating Syndrome (NES) in adults with Type 2 diabetes mellitus (T2DM) [4]. They found that BED and NES are common among adults with T2DM, and that BED is associated with higher BMI in these patients. Moskovich and colleagues performed assessments of affect using a real-time telephone-based survey system among patients with T1DM [23]. They found negative affect and distress over their diabetes increased risk for objective binge eating at the upcoming meal. Studies by Wisting’s group evaluated the Diabetes Eating Problems Survey-Revised (DEPS-R) [24] and employed the survey, along with other measures, to address similarities and differences in eating disorder behaviors, depression, and anxiety experienced by males and females with T1DM [25]. Including males is a much needed advance. They found that worse glucose control, reflected by a higher A1c, was correlated with a higher DEPS-R score. Finally, different screening methods produce different results regarding rates of eating disorders in this population. The paper by Keane and colleagues examines this issue by using the EDE-Q, considered the gold standard screening questionnaire, and report lower rates than previously reported [26]. This finding adds to the debate over whether validated general screenings, modified screenings, or T1DM specific screening tools are the best approach when trying to identify eating disorders in the population with T1DM.

In summary, this Special Issue highlights much needed next steps to improve knowledge and clinical care for this high risk population with complex needs. For further reading we suggest the 2017 text by Goebel-Fabbri [27] also reviewed in this Special Issue [28]. We look forward to an improved understanding of the management of diabetes concurrent with an eating disorder.

## Data Availability

Not applicable.

## Abbreviations

BMI: Body Mass Index
T1DM: Type 1 Diabetes
